# Computed Tomography Features and Clinicopathological Characteristics of Gastric Sarcomatoid Carcinoma

**DOI:** 10.3389/fonc.2020.01611

**Published:** 2020-08-11

**Authors:** Yi-yang Liu, Pan Liang, Kai-xiang Feng, Kui-sheng Chen, Song-wei Yue, Jiang Ji, Wei-wei Li, Xi-tong Zhao, Jian-bo Gao

**Affiliations:** ^1^Department of Radiology, The First Affiliated Hospital of Zhengzhou University, Zhengzhou, China; ^2^Department of Thyroid Surgery, The First Affiliated Hospital of Zhengzhou University, Zhengzhou, China; ^3^Department of Pathology, The First Affiliated Hospital of Zhengzhou University, Zhengzhou, China; ^4^Department of Radiology, General Hospital, Ningxia Medical University, Yinchuan, China

**Keywords:** sarcomatoid carcinoma, stomach, gastric cancer, tomography, X-ray computed, diagnosis

## Abstract

**Purpose:**

Gastric sarcomatoid carcinoma (GSC) is a very rare malignant tumor. The purpose of this study is to describe the clinical, computed tomography (CT), and pathologic features of GSC to increase awareness of this entity.

**Methods:**

The CT features and clinical data of five patients with pathologically documented GSC were retrospectively analyzed and compared with the corresponding data of gastric adenocarcinoma and lymphoma.

**Results:**

Among the 5 patients, 4 were male, and 1 was female. The median age was 59 years. Of the 5 cases of GSC, 3 were in the gastric fundus and cardia, 1 was in the gastric body, and 1 was in the gastric fundus. The gastric wall had local thickening in 4 cases and mass formation in 1 case, with stenosis and deformation of the adjacent gastric cavity. The long-axis diameter of the lesions ranged from 1.4 to 10.2 cm (mean, 4.97 cm) and was <10 cm in 4 cases and >10 cm in 1 case. The tumor showed predominantly inhomogeneous density, with radiodensity values ranging from 30 to 53 HU. In addition, ulcers with an irregular base and slightly raised borders were observed in 4 of 5 cases. After an injection of contrast material, heterogeneous (*n* = 4) or homogeneous (*n* = 1) enhancement was observed. After contrast medium injection, obvious enhancement was seen in 2 cases, and moderate enhancement was seen in 3 cases; the peak tumor signal was observed in the portal phase. Two of the patients demonstrated evidence of lymph node involvement, and in one patient, the boundary between the lesion and the left lobe of the liver was unclear, with low attenuation in the right lobe of the liver with circular enhancement. The remaining two patients showed no evidence of metastasis.

**Conclusion:**

Although GSC is extremely rare, it should be considered in the differential diagnosis of gastric adenocarcinoma and lymphoma. CT findings, combined with patient age and sex, can provide support for the diagnosis of GSC. However, the final diagnosis must be confirmed with histopathology.

## Introduction

Sarcomatoid carcinomas (SCs) are extremely rare aggressive malignant tumors characterized by distinct cellular morphology (1). The features of this tumor were first described in 1982 by Snover et al. (2). SCs can occur in a wide variety of sites, including the respiratory tract, digestive tract, genitourinary tract, breast and thyroid glands (3). However, these tumors are rare in the digestive tract, especially in the stomach. As of April 2020, there are only six cases of gastric sarcomatoid carcinoma (GSC) reported in the English medical literature. These previous reports focused on the pathological and clinical manifestations; them have not systematically described the radiologic appearance of the tumor. Due to the more invasive nature and poorer prognosis of GSC than pure gastric adenocarcinoma (GAC) and gastric lymphoma (GL), it is clinically beneficial to narrow down the differential diagnoses by understanding the computed tomography (CT) characteristics of GSC. The present study analyzed our experience in diagnosing five patients with GSC in terms of the imaging findings and clinical features. To the best of our knowledge, our study represents the largest series of GSCs to date.

In addition, due to the rarity of GSC, the differential diagnosis between GSC and other types of malignant gastric tumors has not received much attention, so we also initially explored the differential diagnosis of GSC from GAC and GL.

## Materials and Methods

The protocol was approved by the Medical Ethics Committee of Zhengzhou University. Informed consent was obtained from all patients.

### Patient Selection

From August 2010 to January 2020, we searched the pathology records and the Picture Archiving and Communication Systems (PACS) of our hospital. The search terms included (stomach) and (sarcomatoid carcinomas). A total of five patients were identified as having SC and were enrolled in the present study. We retrospectively reviewed all clinical data (demographic features, laboratory findings, clinical interventions) and the histologic findings of the five biopsy or operation specimens.

### CT Evaluation

Five GSC patients underwent CT examinations. The CT scans were acquired with a 64-row multidetector device (DiscoveryCT750HD, GE Healthcare, Waukesha, WI, United States). Conventional axial scanning was performed before and after an intravenous (i.v.) injection of nonionic iohexol (iopromide, 370 mg/mL, GE Medical Systems, 1.5 mL/kg and 3 mL/s) through a dual-head pump injector (Medrad, Warrendale, PA, United States). The imaging parameters were as follows: tube voltage, 120 kV; tube current, 350 mA; field of view (FOV), 500 mm; matrix, 512 × 512 mm; and section thickness, 0.75 mm. Finally, a 20-mL saline flush was performed at a rate of 3 mL/s.

Contrast-enhanced CT scans were acquired with scanning delays of 30 s (arterial phase, AP) and 70 s (portal venous phase, PP) after the i.v. injection of the contrast agent started. The CT dose index volume for the three phases was 15 mSv.

### Image Analysis

Two experienced radiologists, 14 and 30 years of abdominal CT experience, performed a retrospective analysis of the CT images. All analyses were performed with an AW4.7 workstation (GE Healthcare), and the radiologists were blinded to the clinical information of the patients. The evaluated parameters included the tumor location (gastric cardia, gastric fundus, gastric body, gastric angle, and gastric antrum), long-axis diameter, shape, growth pattern, serosa condition, attenuation, and enhancement characteristics, such as the enhancement pattern and degree of enhancement. The enhancement pattern of the tumor was classified as homogeneous or heterogeneous based on the AP image. The degree of enhancement of the tumor was based on dynamic CT imaging using HU attenuation, where “obvious enhancement” was defined as >40 HU, “moderate enhancement” as >20 HU and “mildly enhancement” as <20 HU.

The GSCs were staged with the Union for International Cancer Control (UICC) TNM staging standard. All imaging findings were compared with the postoperative pathological findings. The accuracy rate = the number of CTs coincident with the pathological diagnosis/the number of actual pathological diagnoses × 100%.

### Pathological Evaluation

Three patients underwent gastrectomy, and two underwent endoscopic biopsy. The three gastrectomy specimens measured 23 cm × 14.5 cm × 1.8 cm, 14.0 cm × 7.5 cm × 1.0 cm, and 23 cm × 8 cm × 4 cm, respectively; in two of these tumors, the mucosal surface of the excised specimen showed ulcerative masses of approximately 7.0 cm × 6.0 cm × 1.0 cm and 5 cm × 3 cm. The remaining specimen was a soft mass measuring 13 cm × 10 cm × 2 cm. For biopsy, multiple samples were acquired, and the diameter of each sample was 0.3 cm. According to the relevant literature, the diagnostic criteria for GSC were generally as follows: (1) the tumor originated from the stomach; and (2) the tumor consisted of both carcinomatous and sarcomatoid components, and the sarcomatoid component accounted for more than 50% of the tissue. In addition, if biopsy was performed, the sarcomatoid component can be seen in every sample. Furthermore, sarcomatoid regions express epithelial markers such as CK or EMA.

The specimens were fully stretched, fixed and soaked in 3.7% formaldehyde solution for 24 h. All biopsy specimens were analyzed. The specimens underwent routine dehydration, paraffin embedding, sectioning into 4 μm thick sections, and hematoxylin eosin (HE) staining. Immunohistochemical staining was performed using a Roche BenchMark XT automatic immunohistochemical detector. The antibodies used in this study included AE1/AE3, CK(L), CK8/18, epithelial membrane antigen (EMA), vimentin, P40, P63, and antigen KI67 (Ki-67). All antibodies listed above were purchased from DAKO (Dako, Glostrup, Denmark).

### Comprehensive Comparative Analysis

Each patient with GSC was matched by age (±3 years), year of diagnosis, and sex to four patients with GAC, GL; 20 patients with each disease were retrieved from PACS. Patients with GSC were compared with those with GAC, GL in terms of demographic, clinical and CT characteristics (Table 1).

**TABLE 1:** Comparison between GSC and GAC, GL.

	GSC	GAC	GL
Age (median age, range)	(59, 53–65 years)	(54, 49–67 years)	(52.5, 48–66 years)
Main symptoms			
Epigastric discomfort/pain	3 (60%)	13 (65%)	14 (70%)
Intermittent vomiting	1 (20%)	2 (10%)	2 (10%)
Acute hematemesis/Bloody stool	1 (20%)	3 (15%)	4 (20%)
Dysphagia	0 (0%)	2 (10%)	0 (0%)
Location			
Cardia and Fundus	4 (80%)	8 (40%)	2 (10%)
Body	1 (20%)	6 (30%)	10 (50%)
Antrum	0 (100%)	6 (30%)	8 (40%)
The long-axis diameter (median size, range)	(4.2, 1.4–10.2 cm)	(3.4, 1.3–7.7 cm)	(6.6, 1.2–19.2 cm)
Shape			
Focal thickening	4 (80%)	15 (75%)	12 (60%)
Diffuse thickening	0 (0%)	2 (10%)	7 (35%)
Mass	1 (20%)	3 (15%)	1 (5%)
Serosal surface/bare area			
Clear	1 (20%)	15 (70%)	15 (75%)
Unclear	4 (80%)	5 (30%)	5 (25%)
Ulcers			
Yes	4 (80%)	16 (80%)	8 (40%)
No	1 (20%)	4 (20%)	12 (60%)
Density characteristics			
Heterogeneous	4 (80%)	4 (20%)	5 (25%)
Homogeneous	1 (20%)	16 (80%)	15 (75%)
Enhancement patter			
Heterogeneous	4 (80%)	12 (60%)	13 (65%)
Homogeneous	1 (20%)	8 (40%)	7 (35%)
Lymph node involvement*			
Yes	2 (40%)	9 (45%)	8 (40%)
No	3 (60%)	11 (55%)	12 (60%)
Liver involvement*			
Yes	1 (20%)	0 (0%)	0 (0%)
No	4 (80%)	20 (100%)	20 (100%)
Therapy			
Resection	2 (67%)	17 (85%)	2 (10%)
Chemotherapy	0 (0%)	2 (10%)	16 (80%)
Resection and Chemotherapy	1 (33%)	0 (0%)	0 (0%)
Neoadjuvant chemotherapy	0 (0%)	1 (5%)	0 (0%)
Radiation therapy	0 (0%)	0 (0%)	1 (5%)

## Results

### Patient Characteristics

**FIGURE 1:**
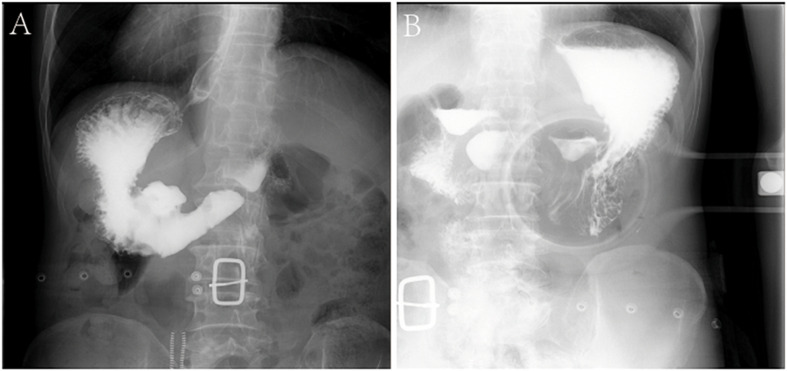
Characteristics of X-ray examinations of a 65-year-old male patient with GSC. **(A,B)** Reveals that there is a huge niche with irregular shapes at the small curvature of the stomach; the niche is located inside the outline of the stomach; the niche is surrounded by transparent bands with different widths, that is, ring embankments, with irregular outlines. The surrounding mucosa is thickened, interrupted, and the local gastric cavity is narrowed.

The patients included four men and one woman ranging in age from 53 to 65 years, with a median age of 59 years. The clinical and CT features of these patients are summarized in Tables 2, 3. All patients had nonspecific symptoms, including abdominal discomfort, epigastric discomfort, nausea or vomiting. The other presenting symptoms included hematemesis or weight loss. Three patients underwent radical resection, in which only one patient was treated with adjuvant chemotherapy after surgery. And two patients chose to deny treatment. In addition, we also reviewed the upper gastrointestinal radiography results (Figure 1).

**TABLE 2:** Clinical and pathological factors of the five GSC patients.

Case	Sex	Age (years)	Complaint	Location	Maximum diameter	Tumor marker (cm)	Anemia	Therapy	Metastasis
1	M	65	Sudden hematemesis	Lesser curvature	5.0	Normal	+	R	+
2	M	59	Epigastric discomfort, Intermittent vomiting	Remnant stomach (Cardia and Fundus)	10.2	TAP (+)	+	Rn	+
3	F	62	Epigastric pain	Cardia and Fundus	4.2	CA125 (+)	+	None	–
4	M	53	Epigastric pain	Fundus	1.4	Normal	–	None	–
5	M	54	Epigastric pain	Cardia and Fundus	4.0	NA	–	R&C	–

*“+” yes/present/positive, “–” no/absent/negative; F = female, M = male, age in years; R = Radical gastrectomy; Rn = Remnant gastrectomy; C = chemotherapy; NA = not available.*

**TABLE 3:** Computed tomography features of the five GSC patients.

Case	Gross features of the tumor	Ulcers	Growth mode	Density characteristics	Enhancement patter	Margin
1	Focal thickening	+	Intracavity	Heterogeneous	Heterogeneous	Unclear
2	Focal thickening	+	Intracavity	Heterogeneous	Heterogeneous	Unclear
3	Mass	–	Intracavity	Homogeneous	Homogeneous	Unclear
4	Focal thickening	+	Intracavity	Heterogeneous	Heterogeneous	Clear
5	Focal thickening	+	Intracavity	Heterogeneous	Heterogeneous	Unclear

*“+” yes/present/positive, “–” no/absent/negative.*

The laboratory findings revealed that patient 2 was positive for tumor abnormal protein (TAP) and patient 3 was positive for carbohydrate antigen 125 (CA125). Before treatment, hemoglobin and erythrocyte count decreased in three patients (patients 1, 2, and 3), and platelet count was elevated in four patients (patients 1, 2, 3 and 4).

### Pathological Features

**FIGURE 2:**
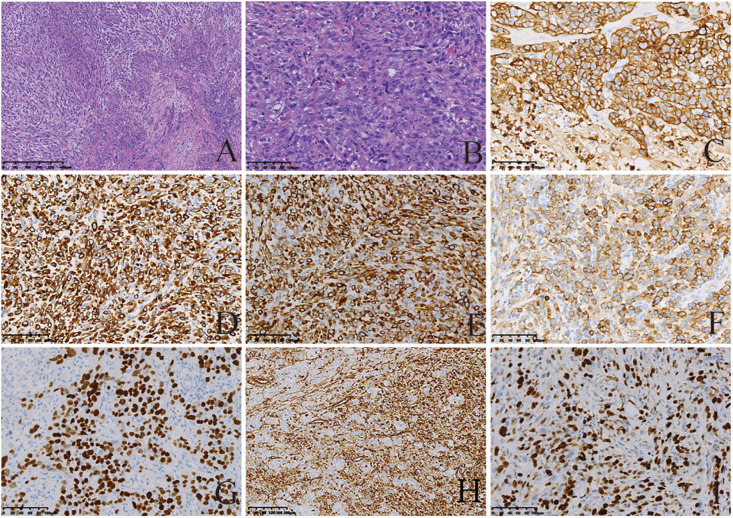
Histological and immunohistochemical features of GSC. **(A,B**) Hematoxylin-eosin (HE) staining showing tumor cells demonstrated spindle-shaped structures, significant atypical nuclei, pleomorphic nuclei and giant nuclei; Mitotic figures visible. Tumor cells showed infiltrative growth. Cells were stained with hematoxylin and eosin stain (magnification, A × 200; B × 50). By immunohistochemistry, the tumor cells were positive for AE1/AE3 **(C)**, CK(L) **(D)**, CK8/18 **(E)**, EMA **(F)**, P40 **(G)**, and vimentin **(H)**. Moreover, 50% of them were positive for Ki-67 **(I)**. The final diagnosis was SC [magnification **(C–I)** ×200].

Micropathologically, the gastric tumor cells showed infiltrative growth. The cytological characteristics of the tumor cells showed obvious malignant characteristics. Microscopically, the spindle cell structure and the nucleus were obviously atypical, pleomorphic and enlarged. Mitotic figures were visible (Figures 2A,B). On immunohistochemical examination, the tumor cells showed positive staining for AE1/AE3, CK(L), CK8/18, EMA, P40, vimentin. The Ki-67 index was higher than 50% (Figures 2C–I). All five tumors were diagnosed as GSC. In addition, the sarcomatoid component showed spindle cell sarcomatoid morphology.

### CT Findings

**FIGURE 3:**
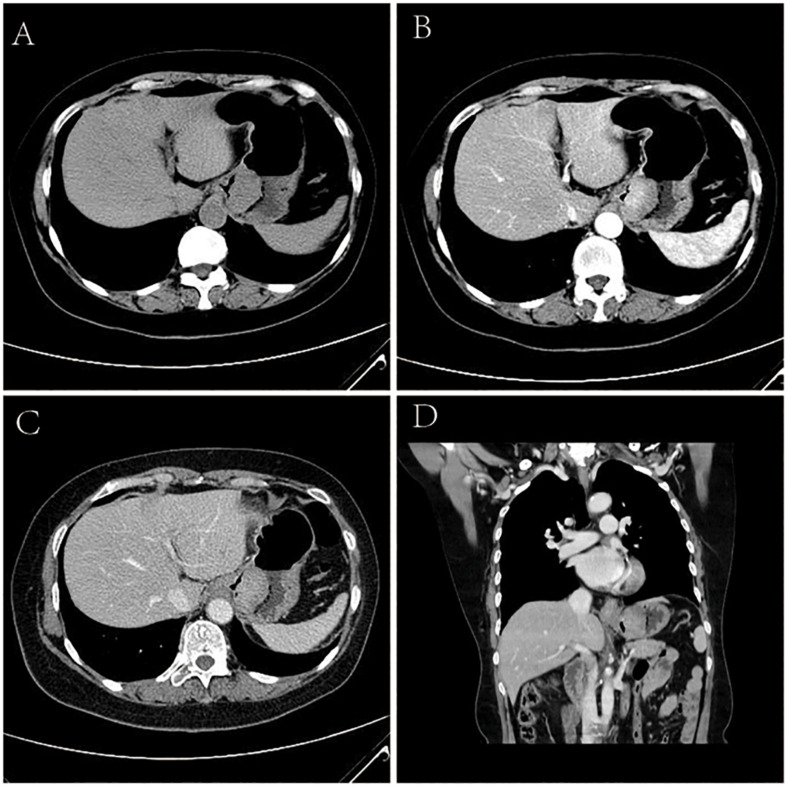
Sarcomatoid carcinoma of the stomach in 62-year-old women. **(A)** Unenhanced CT image of stomach reveals an intraluminal mass of homogeneous attenuation, with an irregular surface, at the gastric fundus and cardiac region. **(B–D)** Contrast-enhanced CT image shows obvious homogeneous enhancement of mass, with the peak value of the tumor on the portal phase. In perigastric lymph nodes, an enlarged and significantly enhancement lymph node can be seen. **(B)** Arterial phase of contrast enhancement image. **(C)** Portal phase of contrast enhancement image. **(D)** Portal phase of contrast enhancement coronal image.

Of the 5 cases of GSC, 3 were in the gastric fundus and cardia (Figure 3), 1 was in the gastric body, and 1 was in the gastric fundus; of these tumors, one was a recurrence in the remnant stomach. The CT manifestations of this tumor included local thickening (*n* = 4), mass formation (*n* = 1). The long-axis diameter of the lesions ranged from 1.4 to 10.2 cm (mean size, 4.97 cm). In addition, ulcers with an irregular base and slightly raised borders were observed in 4 of 5 cases. Among the three patients who underwent surgery, two lesions invaded the gastric serosa, and the remaining lesion invaded the gastric bare area. Among the two patients with biopsy-proven GSC, one patient exhibited tumor invasion of the gastric bare area. The major changes in the CT imaging characteristics were an irregular outer layer of the gastric wall and obscuration of the perigastric fat. Initially, the CT findings were interpreted as GAC in four cases and GL in 1.

The tumor showed predominantly inhomogeneous density, and the radiodensity values were 30–53 HU in the noncontrast phase. Heterogeneous enhancement was seen in four cases due to necrotic or cystic areas, and the other tumor revealed homogeneous enhancement. The radiodensity values on the AP images ranged from 41 to 92 HU and 60 to 96 HU in the venous phase. After contrast medium injection, two tumors showed obvious enhancement, and moderate enhancement was seen in the other three tumors; the peak tumor value was observed in the portal phase. One of the three patients who underwent surgery demonstrated evidence of lymph node involvement; in one patient, the boundary between the lesion and the left lobe of the liver was unclear, and the area with low attenuation was confirmed by pathology as a metastatic lesion in the right lobe of the liver with circular enhancement. The remaining patient showed no evidence of metastasis. Among the two patients with biopsy specimens, one patient was identified as having lymph node metastasis on CT.

### CT Staging Versus Pathological Staging of GSC

None of the GSCs were staged as T1-T2 by CT or pathology. The accuracy of CT staging T3 and T4 GSC was 100% (1/1) and 100% (2/2), respectively. The overall diagnostic accuracy of CT for determining the T stage of GSC was 100% (3/3).

None of the GSCs were staged as N2-N3 by CT or pathology. The accuracy of CT in staging N3 and N4 GSC was 50% (1/2) and 0% (0/1), respectively. The overall diagnostic accuracy of CT for determining the N stage of GSC was 33.3% (1/3).

The comparison of TN staging based on CT and pathology is shown in Table 4.

**TABLE 4:** CT and pathological TN staging for comparison.

Case	CT	Pathological stage
NO. 1	T4aN0	T4aN1
NO. 2	T3N0	T3N0
NO. 3	T3N1	NA
NO. 4	T3N0	NA
NO. 5	T4aN1	T4aN0

*NA = not available. T = tumor; N = node.*

## Discussion

Sarcomatoid carcinoma is an extremely rare and complicated malignant tumor composed of malignant epithelial components and atypical spindle cells. However, the spindle cells of SCs appear to show evidence of epithelial differentiation, for example, showing epithelial markers or epithelial ultrastructural characteristics instead of a specific line of mesenchymal differentiation. Moreover, some of the current literature emphasizes that the sarcomatous components occupy >50% of the elements involved (1, 4). In the present study, our patients’ tumor cells displayed atypical spindle shapes that expressed the epithelial phenotype.

Sarcomatoid carcinomas can occur in almost any organ where carcinoma can occur. In the digestive system, the incidences of SCs in the esophagus and liver are relatively high, but SCs are exceedingly rare in the stomach; we could find only six previous reports in the English literature (Table 5) (1, 4). Between 08/2011 and 4/2020, 753 patients with SC confirmed by pathology were retrospectively analyzed, with only five tumors occurring in the stomach (0.7%). The average age of the reported patients was 62.3 years (range 49–78) and that in our series was 58.6 years (range 53–65). A previous study reported that the sex distribution of male to female GSC patients was 2:1, and the corresponding proportion in our patients was 4:1 (1, 5–7). It has been noticed that SCs are more common in male patients, and sex is a probable risk factor. GSC patients may present with epigastric pain or discomfort, dysphagia, nausea and vomiting, hematemesis, and emaciation. Due to thickening of the gastric wall, pain or discomfort in the upper abdomen is common. The symptoms can last from a few days to several years without obvious specificity.

**TABLE 5:** Clinical and imaging features of six previously reported cases of GCS.

Case	Gender	Age (years)	Location	Size (cm)	Shape	Ulcers	Enhance appearance	Recurrence/Metastasis?	Therapy	Prognosis
1. (6)	M	69	Remnant stomach	20	Polypoid	–	NE	NE	None	NA*
2. (7)	M	78	Greater curvature	5	Polypoid	–	NE	+/–	Surgery	45 Mo. D
3. (7)	F	57	Lesser curvature	5	Polypoid	–	NE	+/–	Surgery	5 Mo. D
4. (7)	F	47	Gastroesophageal junction	5	Ulcerated	+	NE	+/+	Surgery	8 Mo. D
5. (5)	M	74	Remnant stomach	4	Polypoid	+	NE	–/–	Endoscopy	7 Mo. D
6. (1)	M	49	Distal stomach	14	Mass	+	Hyper	–/+	Surgery	2 Mo. D

*The patient succumbed to heart failure before the surgical treatment. An autopsy was performed. “+” yes/present/positive, “–” no/absent/negative. Hyper: hyperdense. NE: no evaluate. Mo = Month, D = Die.

In the present study, 4 of the 5 cases of GSC were recognized in the proximal stomach, and the remaining tumor was found distal to the stomach. Four cases of GSC in the present study had a long-axis diameter less than 10.0 cm, and the remaining tumor had the largest long-axis diameter among our patients (10.2 cm). The location distribution and long-axis diameters of the GSCs in our patients were similar to those in previous reports (1, 5–7).

The diagnosis of SC has always been difficult for clinicians and pathologists, especially the differential diagnosis from carcinosarcoma. Carcinosarcomas are regarded as truly biphasic neoplasms composed of distinct malignant epithelial (carcinomatous) and mesenchymal (sarcomatous) components. The sarcoma components show typical specialized differentiation (8). However, in the actual diagnosis process, the terms “sarcomatoid carcinoma” and “carcinosarcoma” have been used interchangeably in some cases. Therefore, the understanding of these tumors has been hampered. Nevertheless, we can try to focus on whether there is a difference between these tumors from a new perspective. The CT finding SC in the stomach have not been previously scientific reported or even detailed description. There are only four simple descriptions. Chun-Chao et al. (1) reported that a patient with a giant SC presented a mass with a 14 cm diameter in the antrum and body of stomach, which infiltrated the gastric serosa. The hepatic flexure of the colon and gallbladder were also involved on CT. Contrast-enhanced CT images showed obvious enhancement of the two lesions. Sato et al. (5) reported a patient with SC of the remnant stomach, and the radiographic examination showed an elevated lesion with a large ulcer at the gastric cardiac lesser curvature that measured 6 cm in diameter. The other two reports only described a soft tissue mass or a large tumor in the dilated stomach (6, 7). On the other hand, within in the upper gastrointestinal tract, although there are fewer reports of carcinosarcoma localized in the stomach, this type of tumor is still more common than SC (9). Gastric carcinosarcoma showed an elevated lesion or thickened gastric walls in 83%–91% of all reviewed cases (10–12). Tomoaki et al. reported a 79-year-old man with gastric carcinosarcoma, and his veins showed severe invasion. Enhanced abdominal CT showed irregular thickening and slight enhancement of the gastric wall on the side of the lesser curvature, with suspicious bulky lymph nodes (13). Yoshiyuki et al. reported a 70-year-old Japanese woman who presented with a soft tissue mass adjacent to the lesser curvature of the stomach that was lobulated, and CT revealed an ulcer on the lesion. The contrast-enhanced CT images showed heterogeneous enhancement of the mass. The final pathological diagnosis was gastric carcinosarcoma (14). In the present study, we found that GSC showed local thickening of the gastric wall and mass formation, often accompanied by ulcers. The site of the disease was mostly in the proximal part of the stomach, but these tumors can also occur in the remnant stomach. The signal of the tumor was homogeneous or heterogeneous on plain CT scans. After contrast medium injection, 80% (4/5) of tumors demonstrated heterogeneous enhancement on AP images due to cystic areas or necrosis in the lesions. In this study, the enhancement degree of all tumors reached a peak in the PP after contrast enhancement. For these tumors, the enhancement degree in the delayed phase was not significantly reduced. The overall enhancement mode was delayed enhancement. In addition, CT showed that four patients had invasion into the gastric serosal region or gastric bare area, two patients had the characteristics of enlarged perigastric or retroperitoneal lymph nodes and uneven enhancement, and one patient had invasion into the adjacent liver tissue. These findings reflect the metastatic and highly invasive characteristics of GSC. Overall, CT and contrast-enhanced CT can clearly show the primary lesion, infiltration range, lymph node metastasis and distant metastasis of GSC.

Tomographic diagnosis of GSC has not been attempted because of the rarity of this entity. According to the findings of our study, GSC needs to be differentiated from GAC and GL on CT. Adenocarcinoma is the most common pathological type of gastric tumor and is mainly distributed in the antrum, seldomly in the body and fundus of the stomach. The incidence of GAC is high in men, and the median patient age is 67 years (15). The most common CT signs of GAC are local or extensive thickening of the gastric wall, mass formation (including fungoides-type, polypoid-type masses), rough or smooth serous surfaces, and continuous interruption of the mucosal layer. Tumors involving the mucosal surface can appear enhanced 30–35 s after injecting a contrast agent. The peak value for tumors invading the muscular layer usually appears after 60–70 s and after the mucosal surface is strengthened, the duration is longer (16). Primary GL accounts for 1–5% of malignant gastric tumors and is predominantly situated in the gastric antrum, gastric body and gastric fundus. The incidence of GL is high among males, with a median patient age of 55 years. The clinical symptoms included epigastric pain, bleeding, early satiety, and fatigue (17). The most common CT manifestations of GL are diffuse thickening of the gastric wall or a homogeneous soft tissue mass, with slight attenuation or an appearance similar to that of the normal gastric wall. For GL, because of hemorrhage, necrosis, submucosal edema or infarction, the gastric wall may be heterogenous on CT (17). GL originates from a submucosal process, and gastric mucosa is commonly intact in the early stage but shows interruptions or ulceration in the later stage. After contrast medium injection, most GL showed homogeneous and slight enhancement in the delayed phase (17). Lymphoma is considered when distant structures (the mesentery, retroperitoneum, or other parts of the abdomen) have lymph node metastasis (17).

The CT findings may only reflect features of GSC but cannot accurately diagnose GSC, let alone explore the origin of the sarcomatous portion. Immunohistochemistry (IHC) also failed to conclusively establish the origin of GSC. Rodrigues et al. used fluorescence *in situ* hybridization (FISH) to confirm that SC and adenocarcinoma have a common origin, that is, the epithelium (18), while primary GL originated from gastric submucosal lymphoid tissue.

The main treatment for localized lymphomas is eradication of Helicobacter pylori and surgical treatment, whereas advanced disease often requires radiation or chemotherapy alone (19). Surgery is the only treatment option for patients with GAC. Adjuvant chemotherapy and chemoradiotherapy are also often used. Targeted therapy is in the exploration stage (20). However, there are currently no specific National Comprehensive Cancer Network guidelines for the treatment of only GSC because the tumor is relatively rare, although complete surgical resection is the most important treatment method. For example, while chemotherapy is considered in clinical practice, whether chemotherapy can be applied for GSC and the efficacy of chemotherapy remain controversial (1). Domblides et al. first evaluated the efficacy of immune checkpoint inhibitors (ICIs) for SC and found that lung SC patients exhibited high response rates and prolonged overall survival (OS) with ICIs (21). This study provides a new idea for the treatment of GSC.

Because GL tends to be confined to the gastric wall for prolonged periods before tumor spread, its prognosis is better than that of GAC (17). Previous literature has found that SC in the parotid gland, lung, hypopharynx, liver and pancreas have poor prognoses due to metastasis or recurrence, with a survival period of a few months (3, 22–25). Similarly, GSC patients also died or developed metastasis or recurrence within a few months, or it was already in the advanced stage at the first diagnosis. All these clinical manifestations suggest that GSC has a poorer prognosis than GAC and GL (26). In addition, GSC can metastasize through the blood and lymph nodes, and the most common sites of metastasis are the local lymph nodes and liver (5). This conclusion is consistent with our research results.

## Conclusion

The incidence rate of GSC is extremely low, so clinicians and radiologists are not familiar with the features of this tumor. Based on systematic research of this rare tumor and comparisons with common gastric cancers, we found that GSC is more common in men who are approximately 60 years old and is often accompanied by ulcers. The disease is mostly located in the proximal part of the stomach and can also occur in the remnant stomach, with delayed enhancement on contrast-enhanced CT images. These characteristics can provide a reference for further research on GSCs in the future. However, an accurate diagnosis of GSC depends on the combination of clinical, imaging and histopathological features. Due to the aggressive nature and poor prognosis of the tumor, rapid clinical intervention and detailed follow-up with CT are essential.

## Data Availability Statement

The original contributions presented in the study are included in the article/supplementary material, further inquiries can be directed to the corresponding author.

## Ethics Statement

The studies involving human participants were reviewed and approved by the Medical Ethical Committee of the Zhengzhou University. The patients/participants provided their written informed consent to participate in this study. Written informed consent was obtained from the individual(s) for the publication of any potentially identifiable images or data included in this article.

## Author Contributions

YL: manuscript preparation, literature research, and data analysis. PL: literature research and data analysis. KF: manuscript review and data collection. KC: guidance of pathological knowledge. SY: guidance of imaging knowledge. JJ: imaging data collection and analysis. WL and XZ: manuscript editing. JG: study conception and design, manuscript review and guarantor of integrity of the entire study. All authors have read and approved the final manuscript.

## Conflict of Interest

The authors declare that the research was conducted in the absence of any commercial or financial relationships that could be construed as a potential conflict of interest.
